# A low fat diet ameliorates pathology but retains beneficial effects associated with CPT1b knockout in skeletal muscle

**DOI:** 10.1371/journal.pone.0188850

**Published:** 2017-12-14

**Authors:** Jaycob D. Warfel, Bolormaa Vandanmagsar, Shawna E. Wicks, Jingying Zhang, Robert C. Noland, Randall L. Mynatt

**Affiliations:** Pennington Biomedical Research Center, Louisiana State University, Baton Rouge, Louisiana, United States of America; University of Melbourne, AUSTRALIA

## Abstract

Inhibiting fatty acid oxidation is one approach to lowering glucose levels in diabetes. Skeletal muscle specific Carnitine Palmitoyltransferase 1b knockout mice (Cpt1b^m-/-^) comprise a model of impaired fat oxidation; and have decreased fat mass and enhanced glucose disposal and muscle oxidative capacity compared to controls. However, unfavorable effects occur relative to controls when Cpt1b^m-/-^ mice are fed a 25% fat diet, including decreased activity and fat free mass and increased intramuscular lipid and serum myoglobin. In this study we explore if a low fat, high carbohydrate diet can ablate the unfavorable effects while maintaining the favorable phenotype in Cpt1b^m-/-^ mice. Mice were fed either 10% fat (low fat) or 25% fat (chow) diet. Body composition was measured biweekly and indirect calorimetry was performed. Low fat diet abolishes the decreased activity, fat, and fat free mass seen in Cpt1b^m-/-^ mice fed chow diet. Low fat diet also reduces serum myoglobin levels in Cpt1b^m-/-^ mice and diminishes differences in IGF-1 seen between Cpt1b^m-/-^ mice and control mice fed chow diet. Glucose tolerance tests reveal that glucose clearance is improved in Cpt1b^m-/-^ mice relative to controls regardless of diet, and serum analysis shows increased levels of muscle derived FGF21. Electron microscopic analyses and measurements of mRNA transcripts show increased intramuscular lipids, FGF21, mitochondrial and oxidative capacity markers regardless of diet. The favorable metabolic phenotype of Cpt1b^m-/-^ mice therefore remains consistent regardless of diet; and a combination of a low fat diet and pharmacological inhibition of CPT1b may offer remedies to reduce blood glucose.

## Introduction

Considerable evidence supports the idea that oversupply of dietary fat exceeds the storage capacity of adipose tissue and leads to ectopic lipid accumulation resulting in “metabolic stress” in skeletal muscle, liver, pancreas and possibly other tissues, contributing to insulin resistance [1–4]. One prevailing theory is that impaired skeletal muscle fatty acid oxidation (FAO) [5–8] leads to the cytosolic accumulation of fatty acyl-CoAs, diacylglycerol (DAG) and ceramides that are directly linked to defects in insulin signaling [9–14]. Others report lipid oversupply via a high fat diet can actually increase FAO to the extent that carnitine and TCA cycle intermediates are limiting, leading to mitochondrial abnormalities and skeletal muscle insulin resistance [15, 16]. Thus, evidence exists that both lipotoxicity and mitochondrial dysfunction contribute to skeletal muscle insulin resistance. Determining if and how these are intertwined is of great interest, with the fundamentally important question being: Does inhibition of FAO in skeletal muscle contribute to insulin resistance?

Carnitine palmitoyltransferase 1 (Cpt1) catalyzes a rate limiting step of fatty acid oxidation via the shuttling of long chain fatty acids across the mitochondrial membrane. The enzyme is expressed as an isozyme subset, with Cpt1b being the predominant form expressed in skeletal muscle, heart, and brown adipose tissue [2, 4]. Cpt1a is found in brain, intestine, kidney, liver, lung, ovary, pancreas, and spleen [1], and an additional isoform has more recently been discovered, known as Cpt1c, which is predominately expressed in brain tissue [3]. Recent reports have begun to highlight the importance of the muscle isoform, Cpt1b, as a potential pharmacological target, indicating increases in insulin sensitivity upon its inhibition [17]. Furthermore, studies using the pharmacological inhibitors of Cpt1b, etomoxir and oxfenicine, show that these drugs do indeed stimulate increased glucose utilization [5, 18].

While these studies implicate Cpt1b as a potential target for further investigation and development of drugs used to treat type II diabetes mellitus, other studies have demonstrated detrimental side effects in their administration. Widespread inhibition of all Cpt1 isoforms by etomoxir can result in steatohepatitis [7]. Oxfenicine inhibits Cpt1b more selectively [12], but we have shown that ablation of Cpt1b in heart leads to cardiac hypertrophy and increased mortality [19]. These reports have raised questions as to the efficacy of inhibiting Cpt1b specifically in skeletal muscle without affecting the remainder of Cpt1b expressing tissues.

Using a skeletal muscle-specific CPT1b KO model, we showed that mitochondrial FAO inhibition results in a myriad of diabetes risk factors (reduced physical activity; increased circulating non-esterified fatty acids; and increased intramyocellular lipids, diacylglycerols, and ceramides) without inducing insulin resistance [14]. Perhaps more importantly, inhibition of mitochondrial FAO also reprograms muscle metabolism by increasing glucose utilization and increasing mitochondrial number, compensatory peroxisomal fat oxidation, and amino acid catabolism. At the whole body level this reprogramming results in resistance to obesity [14].

More recently we showed that inhibition of mitochondrial FAO induces FGF21 expression specifically in skeletal muscle and that FGF21 acts in a paracrine manner to increase glucose uptake under low insulin conditions and has no effect on adiposity in Cpt1b^m-/-^ mice [13]. Though these changes result in the preservation of efficient usage of carbohydrates in Cpt1b^m-/-^ mice, side effects such as the accumulation of intramyocelluar lipids (IMCL), myodegeneration, and decreased activity and energy expenditure also occur [14].

As Cpt1b continues to develop as an intriguing target for the treatment of metabolic disorders, it is important to consider possible effects of varying dietary intake during such treatment. Known clinical variants of Cpt1b have not been functionally characterized as yet. However, maintenance strategies for individuals with Cpt1a defects involve limiting exercise, fasting, and fat intake, while also ensuring high dietary levels of carbohydrates [11]. We therefore hypothesized that Cpt1b^m-/-^ mice might show fewer negative side effects associated with excessive fat intake when fed a diet low in fat and high in carbohydrates. In the current report we examine the effects of varying diets upon Cpt1b^m-/-^ mice in order to determine the flexibility of their previously observed phenotypes; and to assess optimal diet conditions in systems where Cpt1b action is decreased in an attempt to maintain or even restore insulin sensitivity.

## Materials and methods

### Animal studies

In order to address the role of impaired skeletal muscle FAO in the development of insulin resistance, we targeted the muscle-specific isoform of carnitine palmitoyltransferase, Cpt1b. We crossed mice bearing floxed alleles of *Cpt1b* to *Mlc1f-Cre* transgenic mice for specific ablation of Cpt1b in skeletal muscle, but not cardiac tissue [9]. C57L/B6J albino ES cells were used and transferred to C57L/B6J blastocysts. Details of the *Cpt1b* targeting construct and breeding scheme to generate constitutive muscle-specific inactivation of *Cpt1b* are shown in S1A Fig of Wicks et al [14].

Animal studies were conducted at Pennington Biomedical Research Center’s AALAC-approved facility. All experiments were in compliance with the NIH Guide for the Care and Use of Laboratory Animals, and approved by the Pennington Biomedical Research Center Institutional Animal Care and Use Committee (IACUC). All mice were 3–4 month old male mice on C57BL/6 background unless specified otherwise. Unless otherwise stated, mice were multi-housed, and all were exposed daily to 12 hours of light and 12 hours of dark and fed different diets as indicated (S1 Table). Mice were sacrificed by C0_2_ asphyxiation followed by cervical dislocation, according to approved procedures of the Panel on Euthanasia of the American Veterinary Medical Association.

### Animal procedures

Body composition was measured using a Bruker NMR Minispec (Bruker Corporation). Serum and plasma collections were performed by submandibular bleeding. GTT were performed following a 4-h fast by intraperitoneal injection of 20% D-glucose (40mg of glucose per mouse). Behavioral and indirect calorimetry studies were done in a 16-chamber Oxymax system (Columbus Instruments) as described previously [15, 16]. For food intake studies, a modification of previously described procedures was used [20]. Briefly, 8 week old male mice were singly housed in filter-top cages and kept at an ambient temperature of 22–23°C in a specific-pathogen free facility. Several days prior to beginning food intake measurements, bedding was removed and replaced with stainless steel wire floor inserts for recovery of food spillage. 1.5 inch diameter Polyvinylchloride nesting tubes were provided to reduce time on wire flooring.

### ELISA

ELISA kits were used for measurement of IGF-1, Myoglobin, and FGF21 (Abcam, Cambridge, MA, USA; Life Diagnostics, West Chester, PA, USA; and BioVendor, Brno, Czech Republic, respectively) in serum. IGF-1 and Myoglobin were tested in the fed state, while FGF21 was assayed in overnight fasted serum.

### Quantitative RT-PCR

Total RNA from red quadriceps muscle was isolated for later qRT-PCR using an RNeasy Mini Kit supplemented with DNase digestion (Qiagen). cDNA was then synthesized with an iScript cDNA synthesis kit and was used for qRT-PCR with the SYBR Green system (Bio-Rad). Analysis was conducted using the ∆∆C_T_ procedure described previously [21]. Quantification of mouse cyclophilin B and 18s transcripts were used in all experiments as controls for normalization of gene expression. Primer details are provided in S2 Table.

### Electron microscopy

Soleus muscles were fixed in 2% (wt/vol) glutaraldehyde/1% (wt/vol) formaldehyde and then postfixed in 2% (wt/vol) osmium tetroxide, en bloc stained in 0.5% uranyl acetate, and embedded in Epon-NMA. 70nm ultrathin sections were mounted on collodion-coated copper grids, stained with Reynolds lead citrate, and imaged with a JEOL 100CX transmission electron microscope at the Socolofsky Microscopy Center at Louisiana State University.

### Statistical analysis

Data are expressed as mean ± s.e.m. For measurement of serum proteins and lipids, blood glucose levels, and activity Microsoft Excel software was used for analysis of variance with paired two-tailed Student’s *t-*tests where normality was established using GraphPad Prism software and the D'Agostino-Pearson normality test. P≤ 0.05 was considered significant. For adiposity, gene expression, and food intake studies populations were not often normally distributed or had sample sizes of N<8. For these analyses GraphPad Prism software and Mann-Whitney U Tests were performed as a measure of significant differences with P≤0.05 considered significant. JMP software from SAS was used for ANCOVA analysis.

## Results

### Increased fat intake leads to decreased gain of fat and fat free mass in Cpt1b^m-/-^ mice

**Fig 1:**
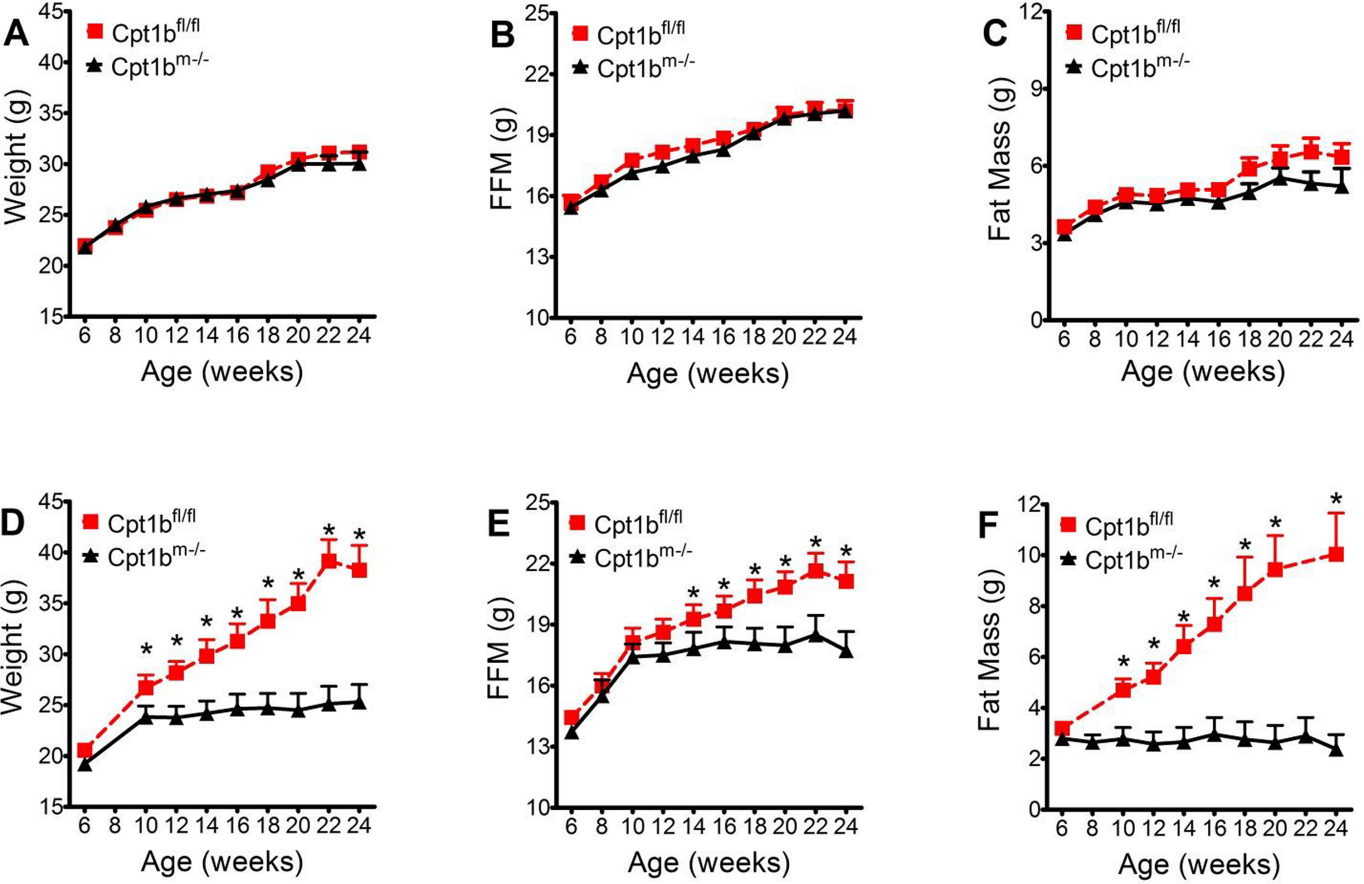
Diet effects on Cpt1b^m-/-^ adiposity. Control mice (red dashed lines, N = 10 animals per time point on chow and N = 8 animals per time point on low fat diet) and Cpt1b^m-/-^ mice (black lines, N = 9 animals per time point on chow and N = 12 animals per time point on low fat diet) were monitored for 24 weeks to assess gain of Weight, Fat, and FFM. Results are compared between groups fed low fat diet (A-C) and chow diet (D-F). Asterisks indicate significance with P ≤ 0.05.

In order to determine if phenotypic differences between Cpt1b^m-/-^ mice and control (Cpt1b^fl/fl^) mice could be ablated by varying nutrient composition within diets, mice were fed low fat (10% fat) or chow (25% fat) diet (S1 Table). As fat content of diet increases, body weight and fat mass also increase in control animals (Fig 1). However, an inverse relation between dietary fat and body mass appears in Cpt1b^m-/- ^mice, where, as fat content of diet increases, body fat and fat free mass decrease (Fig 1). Because these two diets were nearly matched for energy density and protein level, these differences in fat and fat free mass gain can be attributed to the differences in caloric contributions from fats and carbohydrates between the diets.

### Caloric intake and weight gain are restored when Cpt1b^m-/-^ mice switch to low fat diet

**Fig 2:**
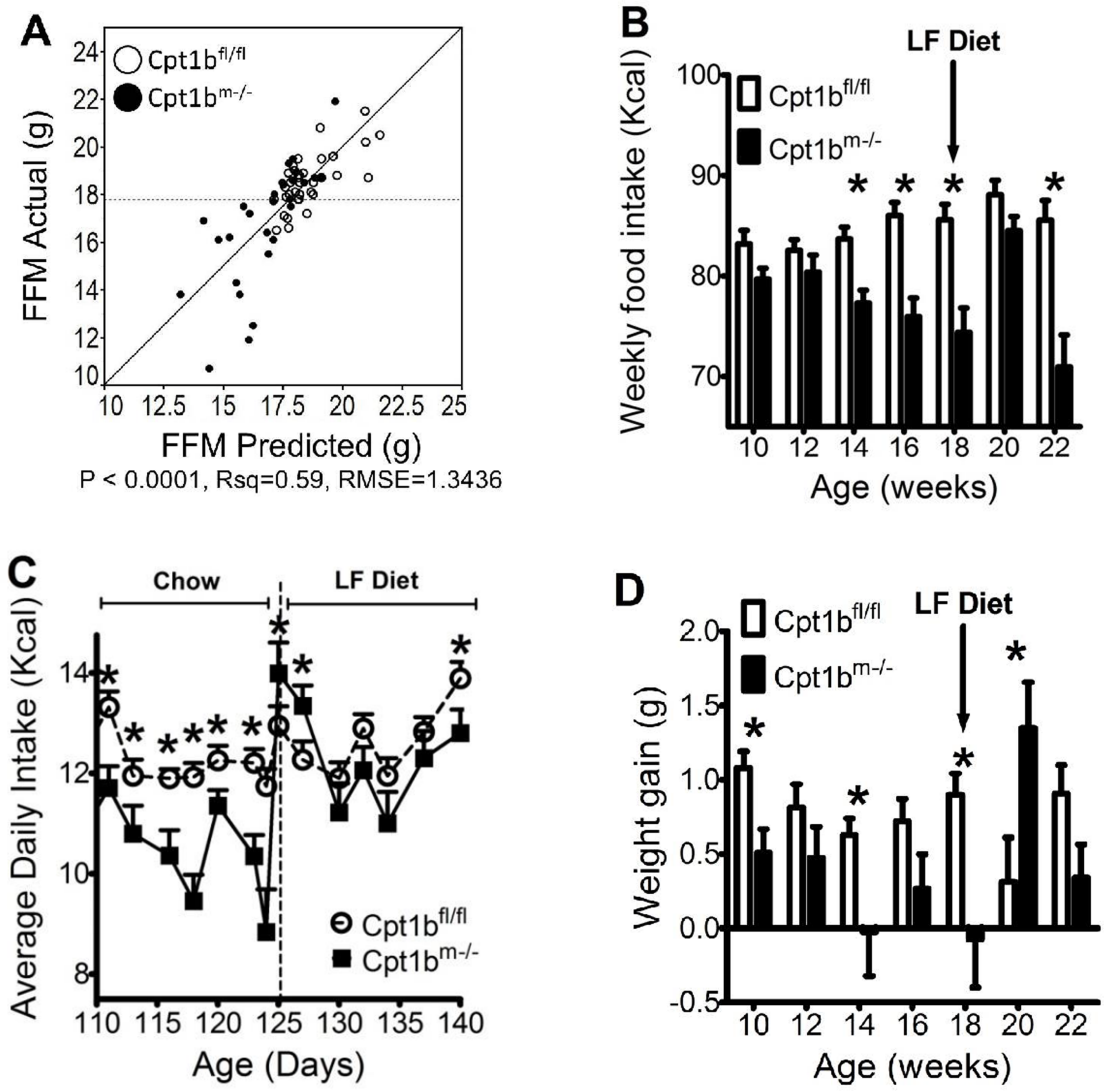
Increased caloric intake as a result of low fat feeding explains increased gain of FFM. (A) ANCOVA analysis conducted by varying FFM as a function of genotype and average daily Kcal intake. White circles represent control mice, while black circles represent Cpt1b^m-/-^ mice. **(**B) Average weekly Kcal intake is plotted for control (white) and Cpt1b^m-/-^ (black) mice during this food switching study. (C) Average daily food intake was monitored for control (white circles) and Cpt1b^m-/-^ (black squares) mice that were fed 25% fat diet for 125 days, followed by switching to low fat diet. (D) Average weekly weight gain during this food switching study is shown for control (white) and Cpt1b^m-/-^ (black) mice. N = 15 animals per genotype for chow diet and N = 10 animals per genotype for low fat diet. Asterisks indicate significance with P ≤ 0.05.

We have reported that the most substantial weight differences between control and Cpt1b^m-/-^ animals are due to differences in fat mass, which begins to deviate several weeks before differences in food intake become significant (Fig 1 and reference [14]). ANCOVA analyses suggest that the differences in FFM vary strongly with average daily Kcal intake. When either weight or fat mass are plotted as a function of average daily Kcal intake while accounting for animal genotype as a covariable, there is a strong genotype effect with a p value of < 0.005 (S1 Fig). If FFM is used in place of either weight or fat mass gain, the genotype specific effect is almost completely diminished, exhibiting a p value of 0.968 (Fig 2A). This suggests that decreased energy consumption and decreased FFM are closely intertwined, and that fat mass is not driving food intake.

To further examine the relationship between food intake and body weight, we examined the effect of switching animals fed Chow diet to low fat after 125 days of age. As observed before [14], Cpt1b^m-/-^ mice begin decreasing food intake by twelve weeks of age and stop gaining weight, while control mice eat more with age and continue to gain weight (Fig 2B). The low fat diet switch resulted in an overall increase in average food intake for Cpt1b^m-/-^ mice, which occurred immediately upon switching diets (Fig 2B and 2C). The increase in food intake was accompanied by a concomitant increase in body weight (Fig 2D). While Cpt1b^m-/-^ mice gain more weight when switched from chow to low fat diet, control animals show a decrease. However, these effects on food intake and weight gain were transient, subsiding after about 4 weeks of low fat feeding (Fig 2B and 2D).

### Levels of IGF1, myoglobin, NEFA, ketones, and activity change in Cpt1b^m-/-^ mice depending upon diet

**Fig 3:**
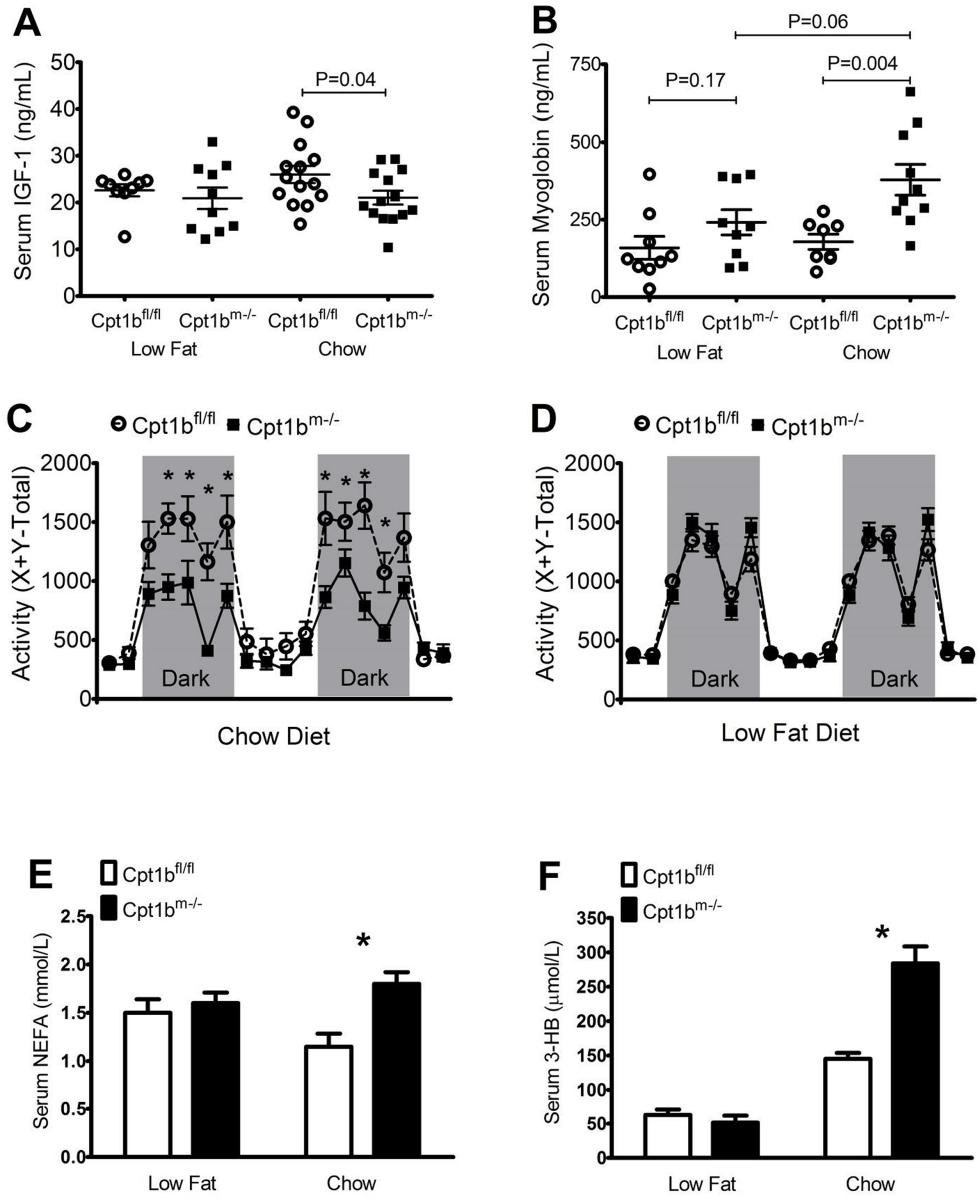
Low fat diet restores levels of IGF-1, myoglobin, activity, and serum lipids in Cpt1b^m-/-^ mice. (A) Serum levels of IGF-1 are plotted for control (white circles) and Cpt1b^m-/-^ (black squares) mice for each of the diets tested at 4–6 months (N = 10 animals per genotype for low fat diet and N = 14 animals per genotype for chow diet). (B) Serum myoglobin levels in animals fed either low fat or chow diets (N = 9 animals per genotype for low fat diet; and N = 10 control and 8 Cpt1b^m-/-^ for chow diet). (C-D) Activity variation is plotted for control (white circles) and Cpt1b^m-/-^ (black squares) mice fed chow diet (C) (N = 9 animals per genotype) or low fat diet (D) (N = 12 animals per genotype). Grey sections indicate periods of darkness, while white sections indicate periods of light. (E-F) Serum levels of NEFA (E) and serum levels of 3-hydroxybutyrate (F) in control (white) and Cpt1b^m-/-^ (black) mice fed chow or low fat diet (N = 9 for control animals and N = 10 for Cpt1b^m-/-^ animals). Asterisks indicate significance with P ≤ 0.05.

Decreased nutrient intake has been shown to decrease body size via lower production of IGF-1 [22, 23]. In line with these findings, we have shown that serum levels of IGF-1 between control and Cpt1b^m-/-^ mice show significant differences when mice eat chow diet [13]. In the present study, serum levels of IGF-1 were also significantly lower in Cpt1b^m-/-^ mice relative to controls when fed chow diet. However, there is no significant difference when mice are fed low fat diet (Fig 3A).

An additional negative characteristic of Cpt1b^m-/-^ mice fed chow diet is a slight loss of muscle function and elevated levels of serum myoglobin relative to controls [14]. Similar results were found in Cpt1b^m-/-^ mice fed low fat diet, although the results become much less statistically significant (Fig 3B). Circulating myoglobin levels are significantly higher in Cpt1b^m-/-^ mice fed chow diet when compared to controls. Feeding the low fat diet, however, lowers myoglobin levels in Cpt1b^m-/-^ mice to the point where they are no longer significantly higher than controls fed the same diet (p = 0.17), but are lower than Cpt1b^m-/-^ mice fed a chow diet with marginal significance (p = 0.06). Cpt1b^m-/-^ mice on a low fat, high carbohydrate diet therefore show a partial correction of the muscle damage observed in Cpt1b^m-/-^ mice eating a chow diet.

In addition to muscle damage, we have previously shown that Cpt1b^m-/-^ mice have reduced activity relative to control mice when fed the 25% fat chow diet [14]. However, control and Cpt1b^m-/-^ mice have similar levels of activity when fed a 10% fat diet (Fig 3C and 3D). Cpt1b^m-/-^ mice also show increases in serum levels of nonesterified fatty acids (NEFA) compared to controls when fed chow diet [14]. This is also true of 3-hydroxybutyrate, indicating an increase in ketogenesis within Cpt1b^m-/-^ mice relative to controls on chow diet (Fig 3E and 3F). Though we see increases in serum lipids of Cpt1b^m-/-^ mice fed chow diet, we have also examined the liver expression levels of genes involved in ketogenesis, fatty acid synthesis and transport, lipid oxidation, glucose oxidation, and gluconeogenesis; and have found no significant increases in any of these genes with the exception of Pepck, indicating a potential increase in gluconeogenesis (data not shown). Much like the serum hormones and activity levels discussed above, the differences seen between genotypes in these lipid metabolites is diminished during low fat feeding. The changes in the levels of these biomarkers and behavioral patterns together with the abolition of weight gain differences suggest that many of the negative physiological effects upon Cpt1b^m-/-^ mice fed chow diet are restored to control levels with low fat diet feeding.

### Diet has little effect on glucose clearance and muscle physiology of Cpt1b^m-/-^ mice

**Fig 4:**
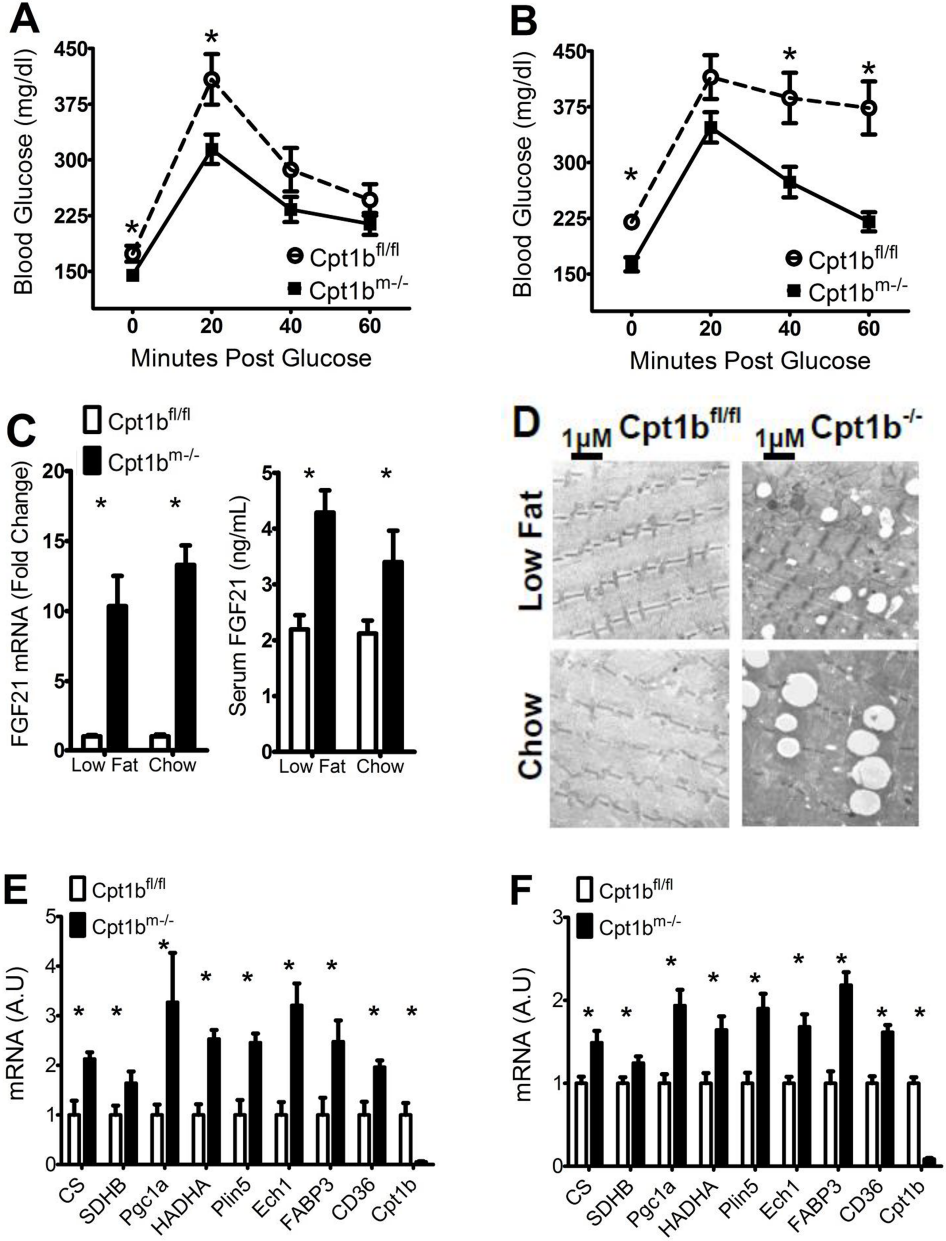
Diet has little effect on glucose clearance and muscle physiology of Cpt1b^m-/-^ mice. (A-B) GTT are shown for control (white circles) and Cpt1b^m-/-^ (black squares) mice that were fed (A) low fat diet (N = 8 animals per genotype) or (B) chow diet (N = 15 animals per genotype). (C) Levels in control (white) and Cpt1b^m-/-^ mice (black) of FGF21 mRNA in red quadriceps muscle (left panel), and FGF21 protein in serum; (right panel) (N = 8 animals per genotype for low fat diet and N = 10 animals per genotype for chow diet). (D) EM showing increased IMCL in soleus of Cpt1b^m-/-^ (right) mice relative to controls (left). (E-F) (N = 4 animals per genotype for low fat diet and N = 6 animals per genotype for chow diet) Relative levels of markers of mitochondrial biogenesis and lipid usage in control (white) and Cpt1b^m-/-^ (black) mouse red quadriceps muscle from mice fed (E) low fat diet or (F) chow diet. Asterisks indicate significance with P ≤ 0.05.

Our previous findings support that Cpt1b^m-/-^ mice have improved glucose clearance relative to control animals when fed chow diet [14]. This finding holds consistent regardless of diet, with blood glucose levels remaining lower in Cpt1b^m-/-^ mice at baseline and throughout the time course of glucose tolerance tests in mice fed either chow or low fat diet (Fig 4A and 4B). For control animals, increased fat content of food predicts increased blood glucose levels; and decreases in blood glucose within sixty minutes post glucose injection are minor relative to Cpt1b^m-/-^ mice. This difference in blood glucose levels is somewhat diminished in animals fed low fat diet, although initial increases in glucose in control animals are significantly larger (Fig 4A). These data suggest that glucose uptake is somewhat enhanced in Cpt1b^m-/-^ mice relative to controls regardless of diet.

One of the adaptations within Cpt1b^m-/-^ mice contributing to enhanced glucose disposal is the increased expression and secretion of FGF21 specifically from skeletal muscle [13]. Regardless of diet, Cpt1b^m-/-^ mice show significantly higher expression and serum levels of FGF21 (Fig 4C). The persistence of this characteristic within Cpt1b^m-/-^ mice relative to controls is likely a factor in their enhanced glucose disposal regardless of dietary composition.

Although Cpt1b^m-/-^ mice have enhanced glucose disposal, they have decreased FAO, and lipid accumulation in skeletal muscle [14]. We therefore sought to test if this phenotype is consistent in both diets. We examined skeletal muscle using electron microscopy (EM) to check for the presence of increased IMCL. As with Cpt1b^m-/-^ mice fed chow diet, those on low fat diet also show markedly increased lipid droplet formation in skeletal muscle over control mice (Fig 3D). Coordinately, the lower fat diet does not affect the upregulation of *Fabp3*, *Cd36*, and *Plin5* genes within Cpt1b^m-/-^ mice fed chow diet, which encode for lipid transport and droplet forming proteins (Fig 4E and 4F). Much like Cpt1b^m-/-^ muscle from mice fed chow diet, muscle from Cpt1b^m-/-^ mice fed low fat diet also display increased levels of markers of mitochondrial FAO such as *Hadha* and *Ech1* (Fig 4E and 4F).

Mitochondrial marker numbers are also enhanced within Cpt1b^m-/-^ muscle, and mitochondria are closely associated with the excessive lipid droplets formed therein [14]. Expression levels of *PGC1α*, an important regulator of mitochondrial number are upregulated relative to control mice within Cpt1b^m-/-^ muscle in mice fed both chow and low fat diets, as are levels of mRNA counterparts of proteins involved in the citric acid cycle and oxidative phosphorylation such as *Cs* and *SdhB* (Fig 4E and 4F). Though only red muscle was used for EM and mRNA analysis (soleus and red quadriceps, respectively), our previous report shows consistency of these results in white muscle as well [14]. To support these findings further, assays of the ratios of mitochondrial genes *Cytb* and *Cox2* to the genomic gene β-globin within white quadriceps are increased roughly 1.5 fold in Cpt1b^m-/-^ mice relative to controls (S2 Fig). These results assert that many of the physiological changes within skeletal muscle that contribute to a lean phenotype with enhanced glucose disposal persist regardless of dietary fat or carbohydrate intake.

## Discussion

Cpt1b^m-/-^ mice gain weight normally up to 10–12 weeks of age when fed a 25% fat chow diet. Control mice continue to increase food intake with age and gain primarily fat mass. In contrast, Cpt1b^m-/-^ mice eat less with age when fed chow diet, their fat mass stays essentially the same as it was at 10–12 weeks of age throughout life, and FFM peaks around 16 weeks of age (Fig 1). If the amount of fat in the diet is dropped to 10%, control mice do not become obese and Cpt1b^m-/-^ mice actually gain the same amount of fat mass as control mice. These data indicate that there may be an aversion to fat in the diet by Cpt1b^m-/-^ mice, because when switched from the 25% fat diet to the 10% fat diet Cpt1b^m-/-^ mice have a temporary increase in food intake (Fig 2). However, it should be noted that fat mass becomes significantly different between control and Cpt1b^m-/-^ mice well before food intake becomes significantly different [14]. Such decreases in food intake do not occur until significant differences in FFM emerge, and ANCOVA analysis suggests that FFM levels are driving food intake (Fig 2). Therefore it is possible that Cpt1b^m-/-^ mice do not overeat on higher fat diets.

Cpt1b^m-/-^ mice have improved glucose disposal regardless of the amount of dietary fat, indicating that pharmacological inhibition of CPT1b may be an effective means of treating metabolic disorders hallmarked by insulin resistance and obesity. In addition, the skeletal muscle from these mice resembles that from well-trained athletes [24], with increased mitochondrial biogenesis, oxidative capacity, and IMCL, yet they are hypoactive and have some muscle damage when they are fed even a moderate, 25% fat diet [14]. We show here the effects on Cpt1b^m-/-^ mice of altering the fat and carbohydrate content in diet, and report significant ablation of inactivity and muscle damage by inhibition of skeletal muscle Cpt1b when mice eat a diet with a fat content of 10% (Table 1). Overall, the results of this study indicate that a high carbohydrate, low fat diet can ensure that Cpt1b^m-/-^ mice retain fat, FFM and activity at similar levels relative to control mice, while also displaying a healthier metabolic phenotype with increased glucose disposal.

**Table 1:** Changes in Cpt1b^m-/-^ relative to controls fed chow and low fat diets.

Cpt1b^m-/-^ vs Control		
	Chow Diet	Low Fat Diet
**Muscle Metabolism mRNA**		
Fatty Acid Oxidation Genes	↑	↑
Fatty Acid Transport Genes	↑	↑
Pyruvate Oxidation Genes	↑	↑
Ox Phos Genes	↑	↑
**Whole Body Parameters**		
Glucose Disposal	↑	↑
Body Weight	↓	No Change
Fat Mass	↓	No Change
Fat Free Mass	↓	No Change
Food Intake	↓	No Change
Activity	↓	No Change
**Serum Proteins and Lipids**		
FGF21	↑	↑
IGF-1	↓	No Change
Myoglobin	↑	No Change
Nonesterified Fatty Acids	↑	No Change
3-Hydroxybutyrate	↑	No Change

## Supporting information

S1 Table Diets used in this study. (DOCX)

S2 Table Primer sequences for qRT-PCR. (DOCX)

S1 Fig **ANCOVA analysis of weight (A) and fat mass (B) as a function of covariables genotype and average daily Kcal intake.** White circles indicate controls and black circles indicate Cpt1b^m-/-^ mice (N = 10–15 animals per genotype per diet group).
(DOCX)

S2 Fig Expression ratios of mitochondrial DNA genes Cytb and Cox2 to genomic DNA gene β-Globin in) in Cpt1b^fl/fl^ (white bars) and Cpt1b^m-/-^ (black bars) mice. (N = 6 animals per group).
(DOCX)
