# Supplementary material for: A low fat diet ameliorates pathology but retains beneficial effects associated with CPT1b knockout in skeletal muscle
Source: PLoS One. 2017 Dec 14;12(12):e0188850. doi: 10.1371/journal.pone.0188850 (PMC5730174; doi:10.1371/journal.pone.0188850)
Supplement: S1 Table — (DOCX) [file pone.0188850.s001.docx]

**S1 Table: Diets used in this Study**

| Fat Content | Carbohydrate Content | Protein Content | Total Kcal/gram | Source |
| --- | --- | --- | --- | --- |
| 10% Kcal | 70% Kcal | 20% Kcal | 3.85 | Research  Diets ® D12450 |
| 26% Kcal | 54% Kcal | 20% Kcal | 3.83 | LabDiet ® 5015 |
|  |  |  |  |  |
