# Supplementary material for: A low fat diet ameliorates pathology but retains beneficial effects associated with CPT1b knockout in skeletal muscle
Source: PLoS One. 2017 Dec 14;12(12):e0188850. doi: 10.1371/journal.pone.0188850 (PMC5730174; doi:10.1371/journal.pone.0188850)
Supplement: S2 Table — (DOCX) [file pone.0188850.s002.docx]

**S2 Table: Primer Sequences for qRT-PCR**

| Symbol | Ref.Seq ID | Forward primer | Reverse primer |
| --- | --- | --- | --- |
| *Cd36* | NM_001159555 | GCAAAGAACAGCAGCAAAATC | TCCTCGGGGTCCTGAGTTAT |
| *Cpt1b* | NM_009948 | CCCGAGCAGTGCCGGGAAGC | GAAATGAGCCAGCTGTAGGG |
| *Cs* | NM_026444 | CGGGAGGGCAGCAGTATCGG | ACCACCCTCATGGTCACTATGGATG |
| *Ech1* | NM_016772 | TCGCTACTGCACTCAGGATG | AGCAGCCAAGCCCATATCTA |
| *Fabp3* | NM_010174 | GACGAGGTGACAGCAGATGA | CTGCACATGGATGAGTTTGC |
| *Hadha* | NM_178878 | TGACGCTGGTTATCTTGCTG | ATCAGGGCCTTCGATTCTTT |
| *PGC1α* | NM_008904 | AGCCTCTTTGCCCAGATCTTC | CCATCTGTCAGTGCATCAAATGA |
| *Plin5* | NM_001077348 | CTTCCTGCCCATGACTGAG | ACCCCAGACGCACAAAGTAG |
| *Ppib* | NM_011149 | TCCATCGTGTCATCAAGGACTT | CTCATCTGGGAAGCGCTCA |
| *Sdhb* | NM_023374 | GGAGGGCAAGCAACAGTATC | GCACACAGGATGCACTCGTA |
| *18s* | NR_003278.3 | agtccctgccctttgtacaca | gatccgagggcctcactaaac |
