# Supplementary material for: A low fat diet ameliorates pathology but retains beneficial effects associated with CPT1b knockout in skeletal muscle
Source: PLoS One. 2017 Dec 14;12(12):e0188850. doi: 10.1371/journal.pone.0188850 (PMC5730174; doi:10.1371/journal.pone.0188850)
Supplement: S1 Fig — ANCOVA analysis of weight (A) and fat mass (B) as a function of covariables genotype and average daily Kcal intake. White circles indicate controls and black circles indicate Cpt1bm-/- mice (N = 10–15 animals per genotype per diet group). (DOCX) [file pone.0188850.s003.docx]

**
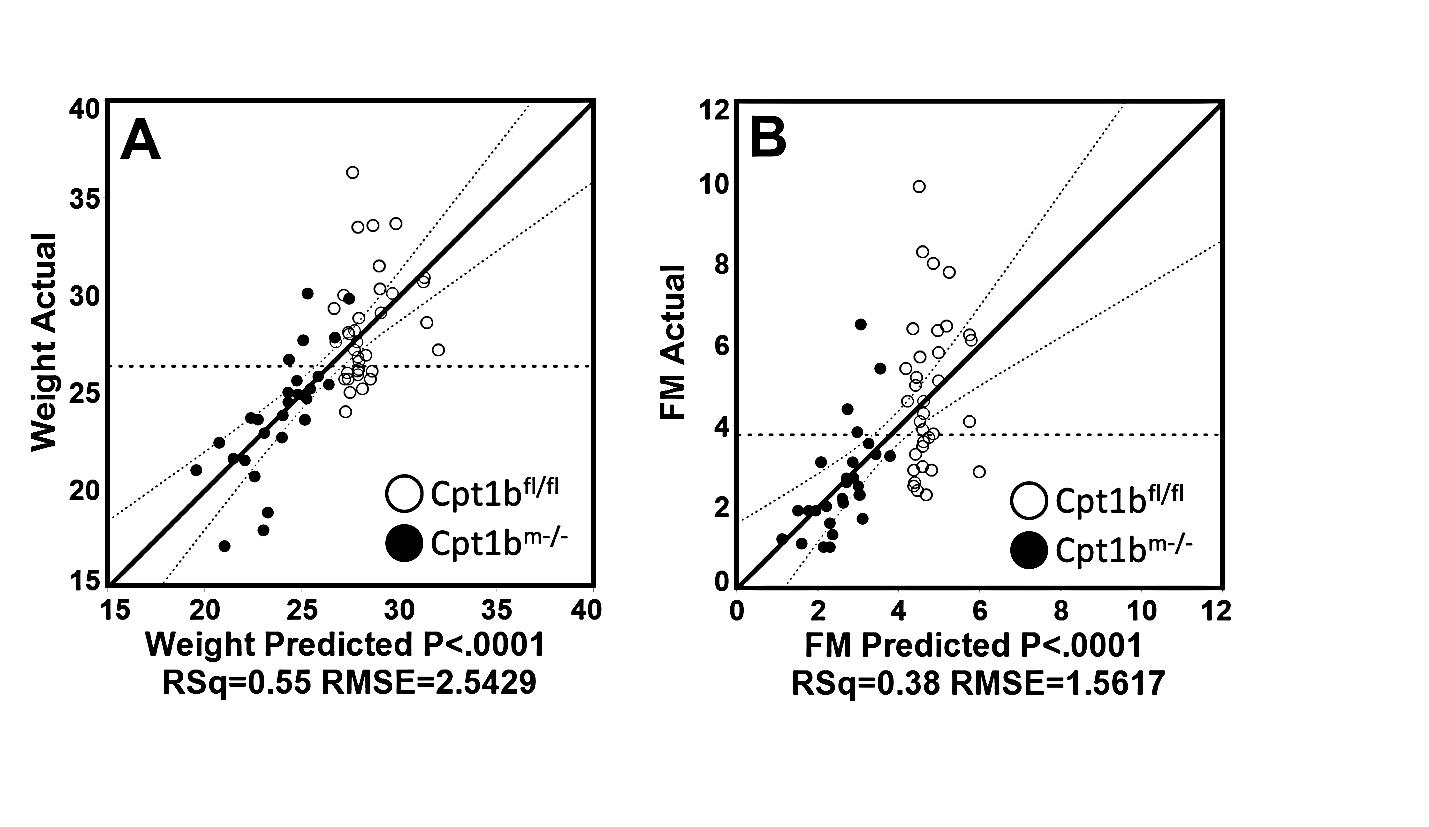
S1 Fig. ANCOVA analysis of Weight (A) and Fat mass (B) as a function of covariables genotype and average daily Kcal intake.** White circles indicate controls and black circles indicate Cpt1b^m-/-^ mice (N=10-15 animals per genotype per diet group).
