# Supplementary material for: A low fat diet ameliorates pathology but retains beneficial effects associated with CPT1b knockout in skeletal muscle
Source: PLoS One. 2017 Dec 14;12(12):e0188850. doi: 10.1371/journal.pone.0188850 (PMC5730174; doi:10.1371/journal.pone.0188850)

**S2 Fig. Expression ratios of mitochondrial DNA genes Cytb and Cox2 to genomic DNA gene β-Globin in ) in Cpt1b^fl/fl^ (white bars) and Cpt1b^m-/-^ (black bars) mice.** (N=6 animals per group).


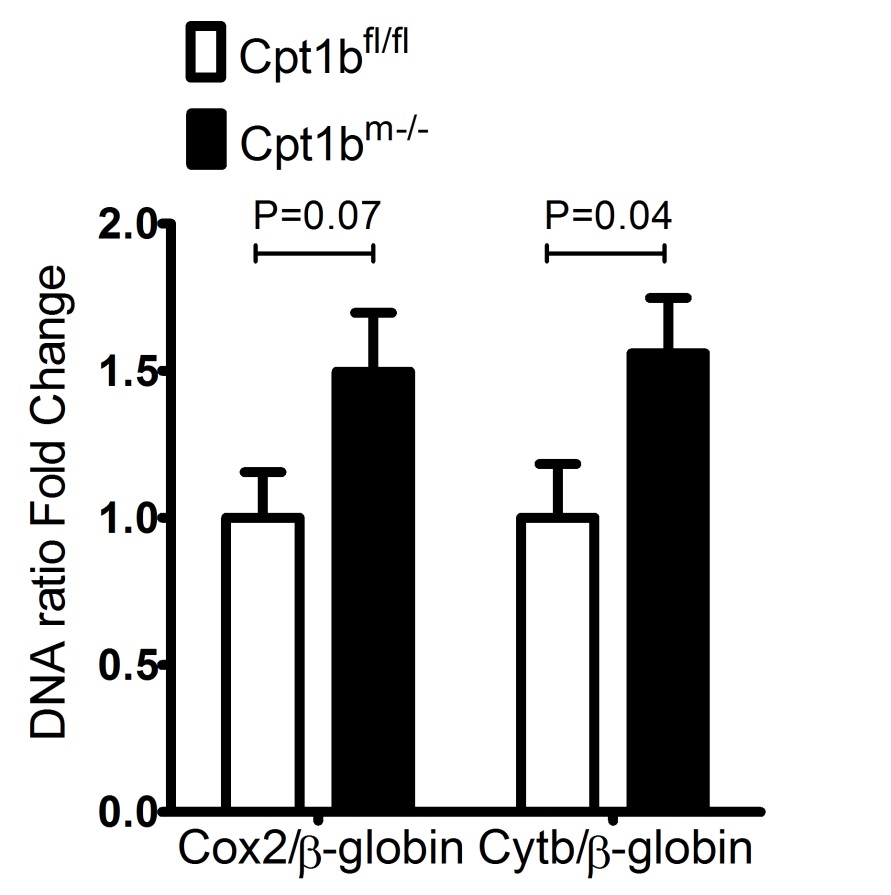

Supplement: S2 Fig — (N = 6 animals per group). (DOCX) [file pone.0188850.s004.docx]
